# Influence of In-Gap States on the Formation of Two-Dimensional Election Gas at ABO_3_/SrTiO_3_ Interfaces

**DOI:** 10.1038/s41598-017-18583-5

**Published:** 2018-01-09

**Authors:** Cheng-Jian Li, Hong-Xia Xue, Guo-Liang Qu, Sheng-Chun Shen, Yan-Peng Hong, Xin-Xin Wang, Ming-rui Liu, Wei-min Jiang, Petre Badica, Lin He, Rui-Fen Dou, Chang-Min Xiong, Wei-ming Lü, Jia-Cai Nie

**Affiliations:** 1 Department of Physics, Beijing Normal University, Beijing, 100875 China; 2 National Institute of Materials Physics, Atomistilor 405A, Magurele, Ilfov 077125 Romania; 3 Condensed Matter Science and Technology Institute, Harbin Institute of Technology, Harbin, 150001 China

## Abstract

We explored in-gap states (IGSs) in perovskite oxide heterojunction films. We report that IGSs in these films play a crucial role in determining the formation and properties of interfacial two-dimensional electron gas (2DEG). We report that electron trapping by IGSs opposes charge transfer from the film to the interface. The IGS in films yielded insulating interfaces with polar discontinuity and explained low interface carrier density of conducting interfaces. An ion trapping model was proposed to explain the physics of the IGSs and some experimental findings, such as the unexpected formation of 2DEG at the initially insulating LaCrO_3_/SrTiO_3_ interface and the influence of substitution layers on 2DEG.

## Introduction

The discovery of two-dimensional electron gas (2DEG) at perovskite oxide interfaces paved the way to observations of new phenomena. For example, the LaAlO_3_/SrTiO_3_ (LAO/STO) interface^1^ has been shown to exhibit two-dimensional (2D) superconductivity^2–5^, ferromagnetism^6–10^, as well as coexistence of these two phases^11–13^. Despite more than a decade of extensive research, the mechanism of 2DEG formation is still debated^14^. The polar catastrophe model^1,15^ is the most widely accepted model (Fig. S1). This model postulates that LAO is polar, with alternating sheets of positive and negative charge in the (001) direction, while STO is not polar. To accommodate the diverging potential caused by the polar discontinuity at the LAO/STO interface, about one half of the electrons and holes accumulate at the interface, forming the 2DEG. This model successfully explains the origin of 2DEG and why the 2DEG at the n-type LAO/STO interface requires a critical thickness of 4 unit cells (u.c.) of LAO while STO has to be TiO_2_-terminated. However, p-type interfaces^1^ and some n-type interfaces are not conductive, (e.g., LaCrO_3_/STO^16^ and LaMnO_3_/STO^17^). Furthermore, the predicted electrical field within the critical thickness is absent^18,19^, and the observed interface carrier density (*n*_*s*_) at the LAO/STO interface is much lower (<10%) than the theoretical value^20^.

Although significant progress has been made in explaining the formation of 2DEG at some interfaces, little attention has been devoted to insulating interfaces with polar discontinuity. The formation mechanism of insulating interfaces must play a vital role in the 2DEG formation and is as important as the polar catastrophe model. Characterization of this mechanism is important for successful formulation of the 2DEG formation theory.

The in-gap state (IGS) is a promising candidate mechanism to fill this gap in the physical understanding. IGSs can absorb the electrons that transfer to the STO conduction band and form 2DEG in the polar catastrophe model. Thus, IGSs can yield a weak electric field in films, a low *n*_*s*_, and even an insulating interface. Current research on IGSs focuses on IGSs in substrates, which are generated by Ti ions in the near-interfacial STO^21–23^. These states can be found at both insulating and conducting LAO/STO^21,23^ interfaces. However, these substrate IGSs cannot explain the insulation properties of interfaces with polar discontinuity, because the difference between these STO-based interfaces is in their films, rather than substrates. Therefore, it is conceivable that IGSs in films play a role in the formation of interfacial 2DEG. In this study, we thoroughly explored film IGSs both experimentally and theoretically, and argued that, combining our results with the polar catastrophe model, a relatively complete theory can be achieved.

## Results and Discussions

### The 2DEG at LaCrO_3_/SrTiO_3_ interfaces

**Figure 1:**
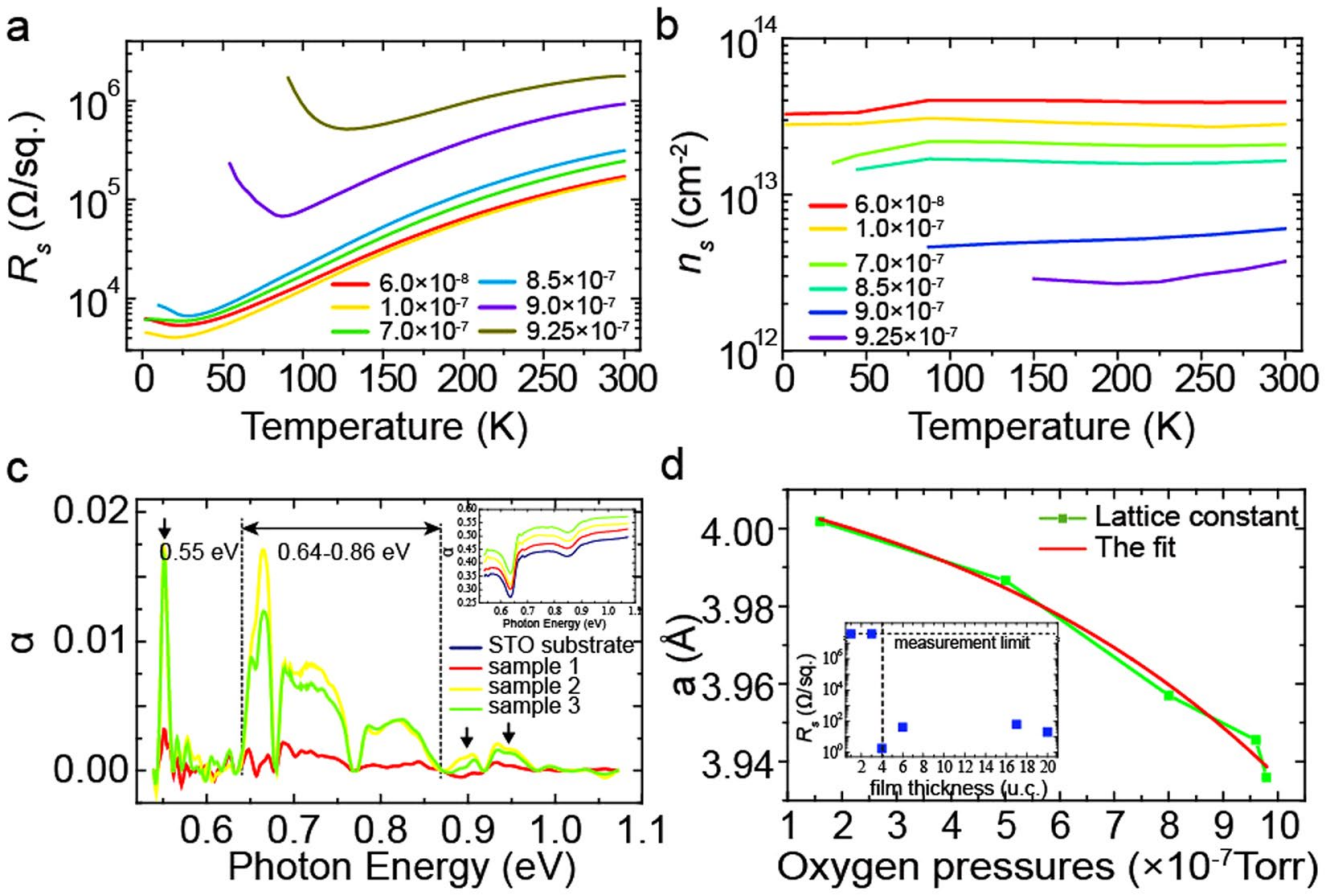
Properties of LCO/STO heterojunctions. (**a**) Interfacial resistance-temperature curves for LCO/STO heterojunctions grown at different *P*_*ox*_ (Torr). (**b**) Interfacial carrier density-temperature curves for LCO/STO heterojunctions grown at different *P*_*ox*_ (Torr). (**c**) Absorption coefficients (α) of different LCO films, after subtracting the influence of STO. The red and yellow curves are the data for 2 u.c. (sample 1) and 17 u.c. (sample 2) LCO films grown at low *P*_*ox*_ (7 × 10^−7^ Torr), and the green curve is the data for 30 u.c. LCO films (sample 3) grown at high *P*_*ox*_ (4 × 10^−6^ Torr). **(d)** The lattice constant of the LCO film grown at different oxygen partial pressures, calculated from XRD data (see methods). The illustration in (**d**) is the interface resistance of LCO/STO with different LCO thicknesses when grown in 2 × 10^−7^ Torr. The critical thickness of 2DEG formation is 4 u.c.

**Figure 2:**
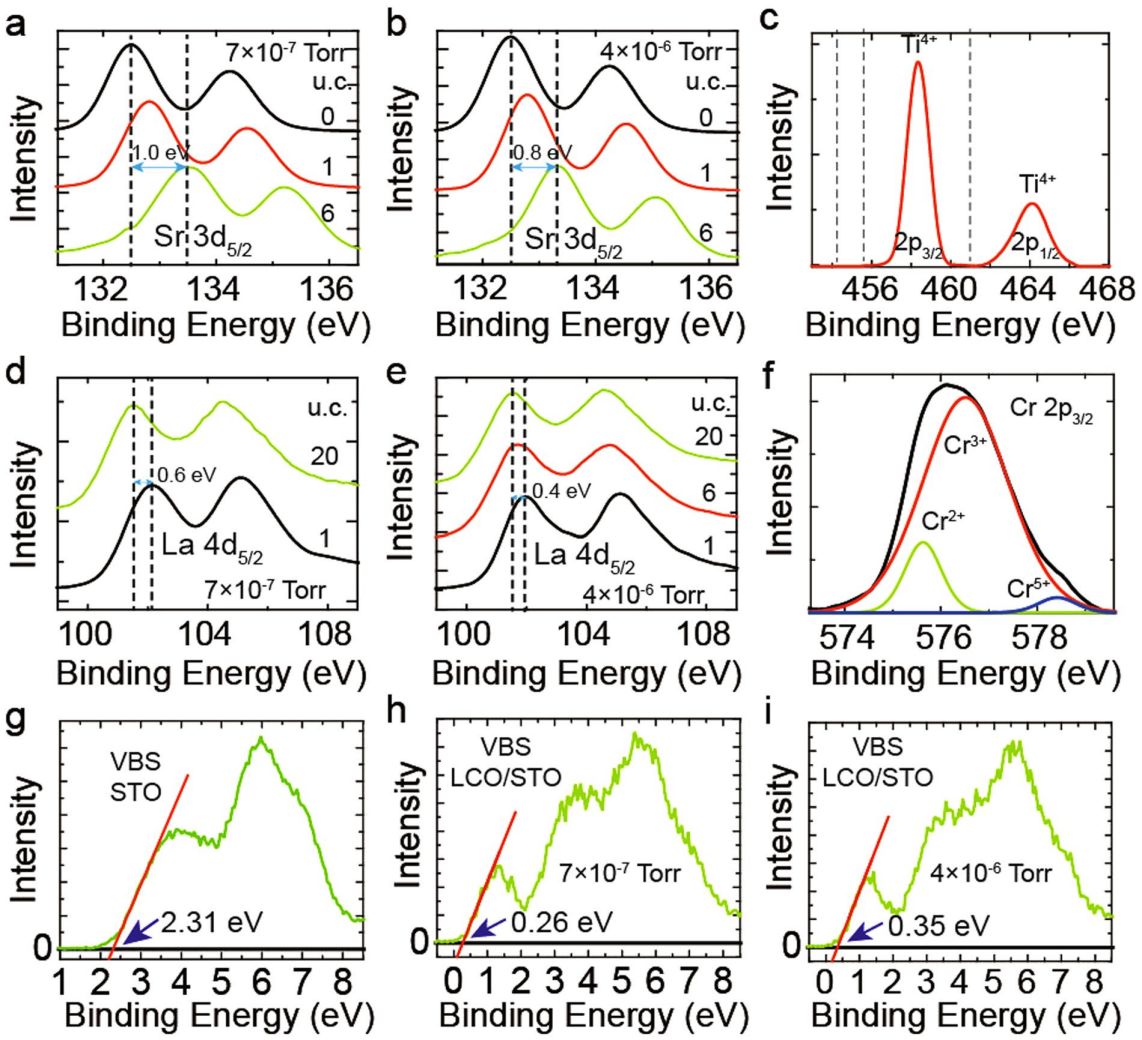
The XPS data. The core level spectra of Sr 3d_5/2_ (**a**,**b**) and La 4d_5/2_ (**d**,**e**) for LCO/STO with different films thicknesses when the films were grown at a low *P*_*ox*_ (**a**,**d**) and high *P*_*ox*_ (**b**,**e**). The LCO(0 u.c.)/STO sample in (**a**) and (**b**) is bare STO. (**c**) The core level spectra of Ti 2p_3/2_ of LCO(1 u.c.)/STO (grown in 7 × 10^−7^ Torr). The black dashed lines are the binding energy of Ti^3+^ if exist. (**f**) The core level spectra of Cr 2p_3/2_ of LCO(20 u.c.)/STO (grown in 7 × 10^−7^ Torr). (**g**–**i**) The VBS of STO (**g**), LCO(20 u.c.)/STO grown in 7 × 10^−7^ Torr (**h**) and LCO(20 u.c.)/STO grown in 4 × 10^−6^ Torr (**i**).

To explore the physical processes yielding insulating interfaces, we grew LaCrO_3_ (LCO) films on TiO_2_-terminated (001) STO substrates, at different oxygen partial pressures (*P*_*ox*_), ranging from 6 × 10^−8^ to 1 × 10^−6^ Torr, using pulsed laser deposition (seen in sample growth). Previously, no 2DEG was observed at the LCO/STO interface^16,24^. To our surprise, 2DEG formed at the LCO/STO interface when the samples were grown at a very low *P*_*ox*_, under 9.0 × 10^−7^ Torr (Fig. 1(a)), and the STO substrates were insulating in all cases. The critical thickness for the 2DEG formation was also 4 u.c. (the illustration in Fig. 1(d)) and the *n*_*s*_ of LCO/STO is similar with the previous reports on LAO/STO^20^ (Fig. 1(b)). At the same time, the films themselves exhibited different behaviors, i.e., in some samples they were semiconducting, owing to the high densities of oxygen vacancies (*n*_*ox*_) in these films (Fig. S2(b)). X-ray photoelectron spectroscopy (XPS) measurements show a high percentage (~12%) of Cr^2+^ (Fig. 2(f)) and indicate a high *n*_*ox*_ in LCO film. The Cr^5+^ cations resulted from the oxidation of Cr^3+^ in the near-surface region of LCO when the sample was exposed to air before the XPS measurement^25,26^. X-ray diffractometry (XRD) measurements indicated that the lattice constant of LCO obviously increased when the films were grown at a lower *P*_*ox*_ (Fig. 1(d)). That is owing to the high *n*_*ox*_ in LCO films^27,28^ and the formation of Cr^2+^ cations (Fig. 2(f)) which has a bigger radius^29^ than Cr^3+^.

The oxygen vacancies in STO substrate may dominate the conduction of the interface due to the low *P*_*ox*_^30,31^. To explore the influence of the oxygen vacancies, we annealed some samples with 2DEG in pure oxygen of one atmospheric pressure for 4 hours (400 °C); after annealing, 2DEG still existed. We also measured the valence of Ti cations of LCO(1 u.c.)/STO grown in 7 × 10^−7^ Torr (Fig. 2(c)) using XPS. To avoid the influence of the polar catastrophe model (the critical thickness of charge transfer is 4 u.c.^1,15^), the LCO thickness is only 1 u.c. We found there are almost no Ti^3+^ signals. That means *n*_*ox*_ in STO substrate should be very low. In addition, we found that the temperature dependence of *n*_*s*_ of the LCO/STO interface is different from oxygen-defects induced conduction of STO^32^ (Fig. 1(b)) and the critical thickness of 2DEG formation (4 u.c.) cannot be explained by oxygen defects. These findings suggest that oxygen defects may be not the main mechanism of 2DEG formation at the LCO/STO interface. For a more in-depth investigation, we grew LaMnO_3_ LaCoO_3_ and STO films on STO, under the same growth conditions as those of LCO (the *P*_*ox*_ was 6 × 10^−8^ Torr), and observed no conducting interface. As we know, oxygen outwards diffusion is film dependent, and the film will influence the *n*_*ox*_ formed in STO side. Previous reports have revealed that the oxygen migration barriers energy (*E*_*m*_, the energy barriers for hopping) of LCO is larger than *E*_*m*_ of LaMnO_3_, LaCoO_3_ and STO^33,34^. So that, the *n*_*ox*_ in STO side of LCO/STO may be slightly lower than that of LaMnO_3_/STO LaCoO_3_/STO. We also annealed the STO substrate under the growth condition of LCO films, at a *P*_*ox*_ of 6 × 10^−8^ Torr, for 2 hours, following which the STO substrate was still found to be insulating. In fact, we found that the STO substrate becomes conductive only when the *P*_*ox*_ is less than 2 × 10^−8^ Torr (annealed at 830 °C for 2 hours). If the LCO/STO interface becomes conducting owing to oxygen defects, the LaMnO_3_/STO LaCoO_3_/STO and STO/STO interfaces, as well as the annealed STO substrate, should all have been conducting; however, this was not the case. Based on these results, we concluded that oxygen defects are necessary but not sufficient to explain the formation of 2DEG at the LCO/STO interface.

### The IGS and band alignment of LCO/STO

We measured the optical transmission spectra of bare STO and of the LCO/STO heterojunctions by a UV-VIS-IR recording spectrophotometer and calculated the absorption coefficients (α) of them (Fig. 1(c)). After subtracting the contribution of STO, we observed several absorption peaks within the band gaps of LCO (1.6 eV^16^) and STO (3.2 eV^14^). These peaks were observed in thick films (regardless the interfacial 2DEG formation), while they were negligible in thin films, suggesting that the peaks are caused by the IGSs in the LCO films, rather than by the substrates or interfaces. We can find sample 3 (30 u.c.) has a lower α than sample 2 (17 u.c.) which is thinner and grown at a lower *P*_*ox*_ than sample 3. If the density of IGSs is same for all samples, sample 3 should have a higher α. This abnormal phenomenon means *P*_*ox*_ or oxygen vacancies may have a great influence on IGSs. Many reports have found that the ions neighboring oxygen vacancies can introduce IGSs, such as the Ti ions in STO^34–37^, the Al and La ion in LAO^36^ and so on. Due to the low *P*_*ox*_, we think the IGSs in the LCO films should result from the La and Cr ions neighboring the oxygen vacancies, and the oxygen vacancies are necessary for the formation of IGSs in films. Because sample 2 was grown at a lower *P*_*ox*_, the density of oxygen vacancies and IGSs should be high than sample 3, so that sample 2 has a higher α.

**Figure 3:**
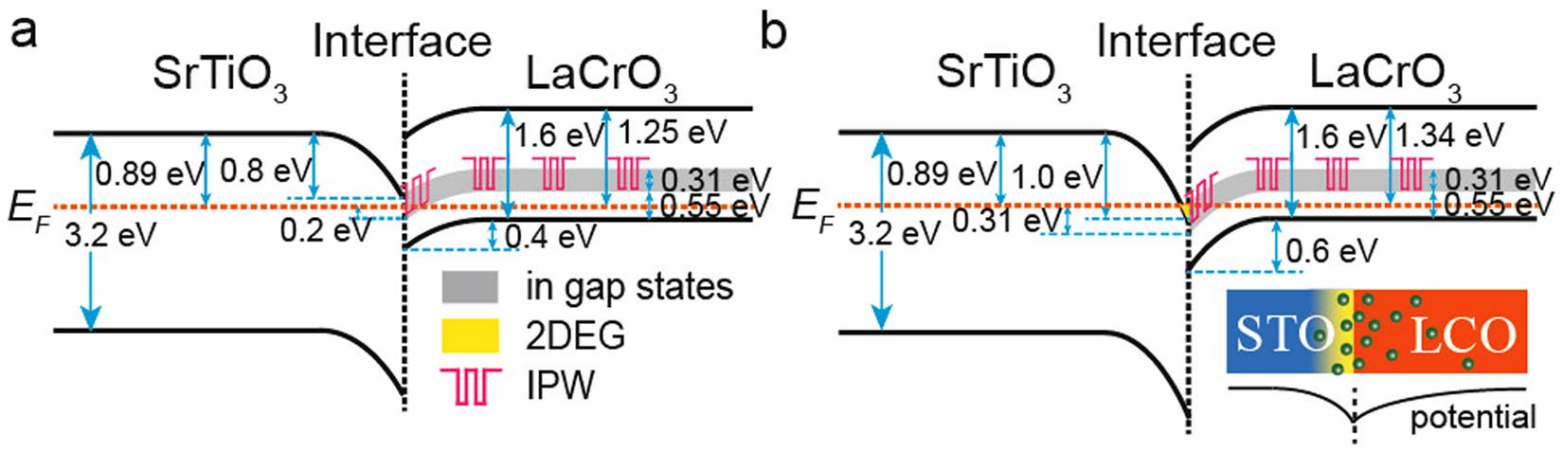
Band alignments of LCO/STO. Band alignment of the LCO/STO interface for a film grown at a high *P*_*ox*_ (**a**) and low *P*_*ox*_ (**b**). Here we only show the IGSs located in the range of 0.55–0.86 eV (Fig. 1(c)). The illustration in (**b**) is the schematic diagram of the electrons distribution (upper row) and the corresponding potential (second row) near the LCO/STO interface. The green balls in the illustration are electrons.

We also measured the band bending in STO substrates and LCO films, using XPS. The core level shift with film thickness changing can be used to quantitatively determine band bending of heterojunctions^14,38^. Here we use the core level shift of A-site cations (Sr 3d_5/2_ and La 4d_5/2_, Fig. 2(a,b,d,e)) to determine the band bending in STO and LCO^16,18,39^. In fact, we found the core level shifts of A-site and B-site cations are very similar (Fig. S2(d–h)). With these data, downward band bending toward the interface was found for both STO and LCO film. The valence band maximum (VBM) of STO and LCO were determined by extrapolating the leading edge of the valence band spectra (VBS) of STO substrate^38,39^ and LCO(20 u.c.)/STO (Fig. 2(g–i)). Because oxygen pressure can influence VBM of LCO (Fig. 2(h,i)), we use 0.26 eV as the VBM of LCO for the LCO films grown in 7 × 10^−7^ Torr (Fig. 2(h)) and use 0.35 eV as the VBM of LCO for the LCO films grown in 4 × 10^−6^ Torr (Fig. 2(i)). Together with the absorption coefficients data, this analysis allowed us to map the band structure of LCO/STO, including the IGSs in the LCO films (Fig. 3).

The band bending on STO side result from the distribution of transferred electrons on STO side^40^ and the mismatch between film and substrate which can lower the energy of *d*_*xy*_ of Ti^7^. The band bending on LCO side can be understood by combining IGSs and the polar catastrophe model. As described in polar catastrophe model, the electric potential caused by the polar-discontinuity at interface should be eliminated by the charge transfer from film to the interface^15^ (Fig. S1(a,b)). However, the IGSs in films can trap a large part of transferred electrons, weaken the charge transfer and make the electric potential cannot be completely eliminated. The residual potential result in a band bending on LCO sides (illustration in Fig. 3(b)).

We can find that when the LCO films were grown at a high *P*_*ox*_ (Fig. 3(b)), the near-interfacial IGSs in these films are below the Fermi energy, while the STO conduction band is higher than the Fermi energy. Thus, electrons are localized in the IGS and could not move to the STO conduction band, preventing the formation of 2DEG. On the other hand, the IGS in the films and the STO conduction band near the interface are both below the Fermi energy when the films were grown at a low *P*_*ox*_ (Fig. 3(a)). As a result, 2DEG formed at the LCO/STO interface, as we have observed (Fig. 1(a,b)). Therefore, because some electrons are localized in the IGSs in these films, *n*_*s*_ should be lower than the theoretical value of the polar catastrophe model, as found previously^20^.

Strangely, we found that there exists a band bending on the STO side when no 2DEG formed at the interface, and the wells on STO side are very deep (Fig. 3). Considering that the band bending on STO side mainly results from the transferred electrons^40^, the former means some electrons still can transfer from film to STO side even when the LCO films were grown in a relatively high *P*_*ox*_. However, because the IGSs in STO can trap a lot of electrons^34–37^, these transferred electrons are localized and the interface is still insulating. We think the latter should result from the oxygen vacancies induced by the low *P*_*ox*_. Firstly, we found the oxygen vacancies can introduce a band bending on STO side (Fig. S2(c)). Secondly, oxygen vacancies can increase the dielectric constant of STO^41^, result in a larger distribution length of electrons on STO side^42,43^ and then enhance the band bending on STO side^40^. As a result of this two reasons, the wells on STO side become very deep.

### The ion trapping model

**Figure 4:**
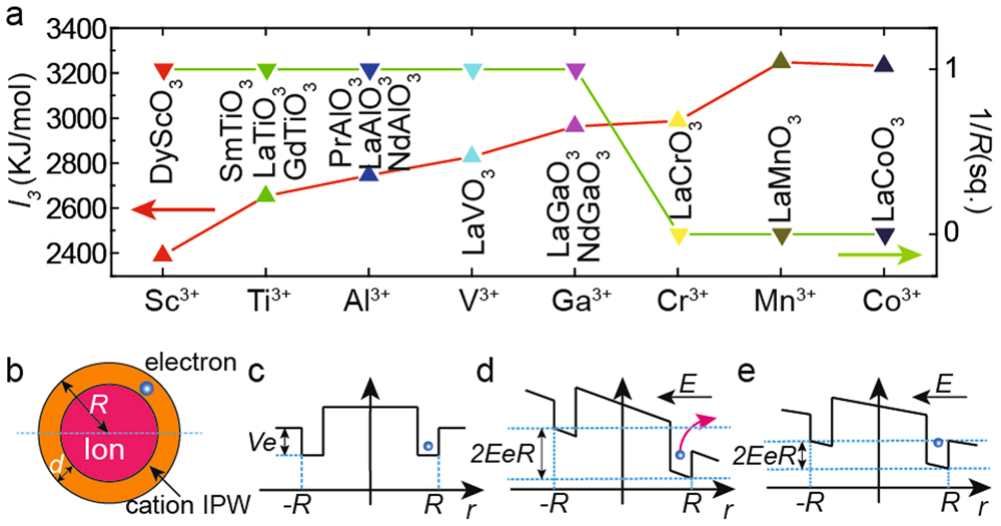
Relationship between 2DEG and ionization energy, and the schematic of IPW. (**a**) The interface electrical conductivity^16,17,44–51^ and the third ionization energy (*I*_3_) of B site cations (Table S1), for different A^3+^B^3+^O_3_/STO heterojunctions have been reported. (**b**) Schematic of the cation IPW; **(c–e)** The *r* dependence of the potential energy of one electron on the position marked by a light blue dashed curve in (**b**) for *E* = 0 (**c**), *2EeR* > *eU* (**d**), and *2EeR* = *eU* (**e**). The situation considering the actual electric field in the polar catastrophe model is shown in Fig. S1.

From the absorption coefficient results, it was very confusing to find that the IGSs in different films are very similar to thick films. Thus, we expect the charge transfer to be affected by the detailed characteristics of IGSs. These characteristics should be material-related and should compete with the charge transfer owing to the polar discontinuity in the polar catastrophe model. To determine these key characteristics of IGSs, we analyzed the A^3+^B^3+^O_3_/STO interface that has been studied previously^16,17,44–51^. We summarized these reports in Fig. 4(a) and found a strong relationship between the 2DEG formation and the third ionization energy (*I*_3_) of the film B site cations. 2DEG can form only if *I*_3_ is less than or equal to 2963 KJ/mol (*I*_3_ of Ga^3+^)^29^. As is known, the ionization energy is the energy required to remove one electron, and it can also be regarded as a propensity to trap an electron by atoms/cations. We suppose that such an electron trapping propensity of cations is the key determinant of IGSs, and cations yield IGSs in films just like substrate Ti cations are at the origin of substrate IGSs^21–23,34–37^. Here, we propose an ion trapping model and try to explain how the IGSs in films work.

Our model utilizes the concept of the ion potential well (IPW), which is the IGS in a film. IPWs are generated by film cations/anions and can trap electrons/holes, preventing them from reaching the interface and thus weakening/destroying 2DEG. The competition between the transfer of charges (polar catastrophe model) and their trapping by ions (ion trapping model) determines the formation of interfacial 2DEG. Different ions have different IPWs, which explains why some interfaces are conductive while others are insulating. We emphasize that a polar system is actually not needed for IPWs, but it is necessary for the formation of 2DEG as described in the polar catastrophe model^1,15^. Our model is simple and does not take into account complex quantum mechanisms. This is somewhat similar to the polar catastrophe model. Our model is a simple try, with it we provide a new and effective way to understand the formation of 2DEG.

Because the trapped charges will move around the ion core and cannot enter into the ion core due to the Coulomb repulsion, we suppose that an IPW is a spherical-shell well with a depth of *eU* (Fig. 4(b)) and a thickness of *d*. We also assume that if the distance between one charge and one IPW is less than the trapping radius *R*, this charge will be trapped by the IPW: this charge will be located within the IPW’s thickness *d*. The trapped charge cannot escape from the IPW unless it is sufficiently energetic. We emphasize that charge transfer is driven by the electric field caused by the polar discontinuity at the interface (*E*_*p*_), as described by the polar catastrophe model. Here, we shall use the cation IPW to explain how our model works (Fig. 4(c–e)). If an electron that is trapped by the cation IPW moves a distance 2 *R* in the direction opposite to that of the electric field (*E*), its energy will increase by 2*EeR*. Here, *e* is the electron charge, and average *E*_*p*_ is the primary part of *E*. For 2*EeR* ≤ *eU*, the electron cannot escape from the IPW, and 2DEG does not form at the interface (Fig. 4(c)). For 2*EeR* > *eU*, the electron can escape from the IPW, moving to the interface (Fig. 4(d)); thus, 2*EeR* > *eU* is the condition for 2DEG formation. The escaped charge generates an additional electric field (*E*_*i*_) and weakens *E*, so that when 2*EeR* = *eU* no more electrons can escape (Fig. 4(e)). 2*EeR* = *eU* is the equilibrium condition between charge transfer and charge trapping.

### Model details

As discussed above, the electric field in polar-non-polar heterojunctions has two main components (Eq. (1)), one owing to polar discontinuity and the other owing to the charges that move to the interface.

1 $$E={E}_{p}+{E}_{i}=\frac{{n}_{p}e}{{\varepsilon }_{r}{\varepsilon }_{0}}-\frac{{n}_{i}e}{{\varepsilon }_{r}{\varepsilon }_{0}}=\frac{({n}_{p}-{n}_{i})e}{{\varepsilon }_{r}{\varepsilon }_{0}}$$

The quantity *ε*_*r*_ is the relative dielectric constant of the film. *n*_*i*_ is the real interface charge density. *n*_*p*_ is the theoretical interfacial charge density in the polar catastrophe model, which is ~3.28 × 10^14^/cm^2^ for A_3_^+^B^3+^O_3_/STO(001) heterojunctions. It is necessary to distinguish between *n*_*i*_ and the real interface carrier density (*n*_*s*_), because not all charges at the interface contribute to conductivity, owing to the IGS in STO^19,22^.

The IPW depth *eU* and the trapping radius *R* are the key factors of the ion trapping model. In our calculations, we assumed that *eU* results from the Coulomb interaction. By a logical extension of the relationship between 2DEG and ionization energy (Fig. 4(a)), the ionization energy of cations or the electronic affinity of anions (*T/ε*_*r*_) can be used as a measure of the trapping ability of cations or anions, the main part of *eU*. Considering the valence of A or B site cations and O^2−^ anions, *T* is the third ionization energy (*I*_3_) for cations, the second electronic affinity (*A*_2_) for O^2−^ anions at the A^3+^B^3+^O_3_/SrTiO_3_ interface (Table S1). On the other hand, the Coulomb potential energy created by all ions in the film also affects *eU*. This potential energy exists everywhere in the film and is the background potential energy (*eU*_0_(*r*)*/ε*_*r*_), where *r* is the charge-IPW distance. It is necessary to subtract *eU*_0_(*r*)*/ε*_*r*_ from *T/ε*_*r*_ to obtain the effective *eU* (Eq. (2)).

2 $$eU=\frac{T}{{\varepsilon }_{r}}-\frac{e{U}_{0}(r)}{{\varepsilon }_{r}}\,if(r\le R)$$

To calculate *eU*_0_, we coded a simple program (see Program details). We found that *eU*_0_(*r*)*/ε*_*r*_ is anisotropic and attains a minimum at *r* = *R* (Fig. S4(f)). Here, we only focus on the [001] orientation and take the maximum value of $eU\,(T/{\varepsilon }_{r}-e{U}_{0}(R)/{\varepsilon }_{r})$ as the effective depth. Thus, *eU* can be regarded as a function of *R*. With the value of *eU*, *2EeR* = *eU* and Eq. (1), we can calculate *n*_*i*_ (Eq. (3)) and the percentage trapping of electrons (*k*) (Eq. (4)) of one type of IPW (see details in *Supplementary Information*).

3 $${n}_{i}=(1-k){n}_{p}$$

4 $$k=\frac{T-e{U}_{0}(R)}{2{e}^{2}R{n}_{p}}{\varepsilon }_{0}\times 100 \% $$

The parameters *k* and *n*_*i*_ are independent of the film thickness or the number of IPWs, resulting in a constant *n*_*s*_, which is in agreement with the experimental values^20^.

2DEG can form only when *k* < 100%, so the condition for 2DEG formation can be written as Eq. (5).

5 $$\frac{2{n}_{p}{e}^{2}R}{{\varepsilon }_{0}} > T-e{U}_{0}(R)$$

**Figure 5:**
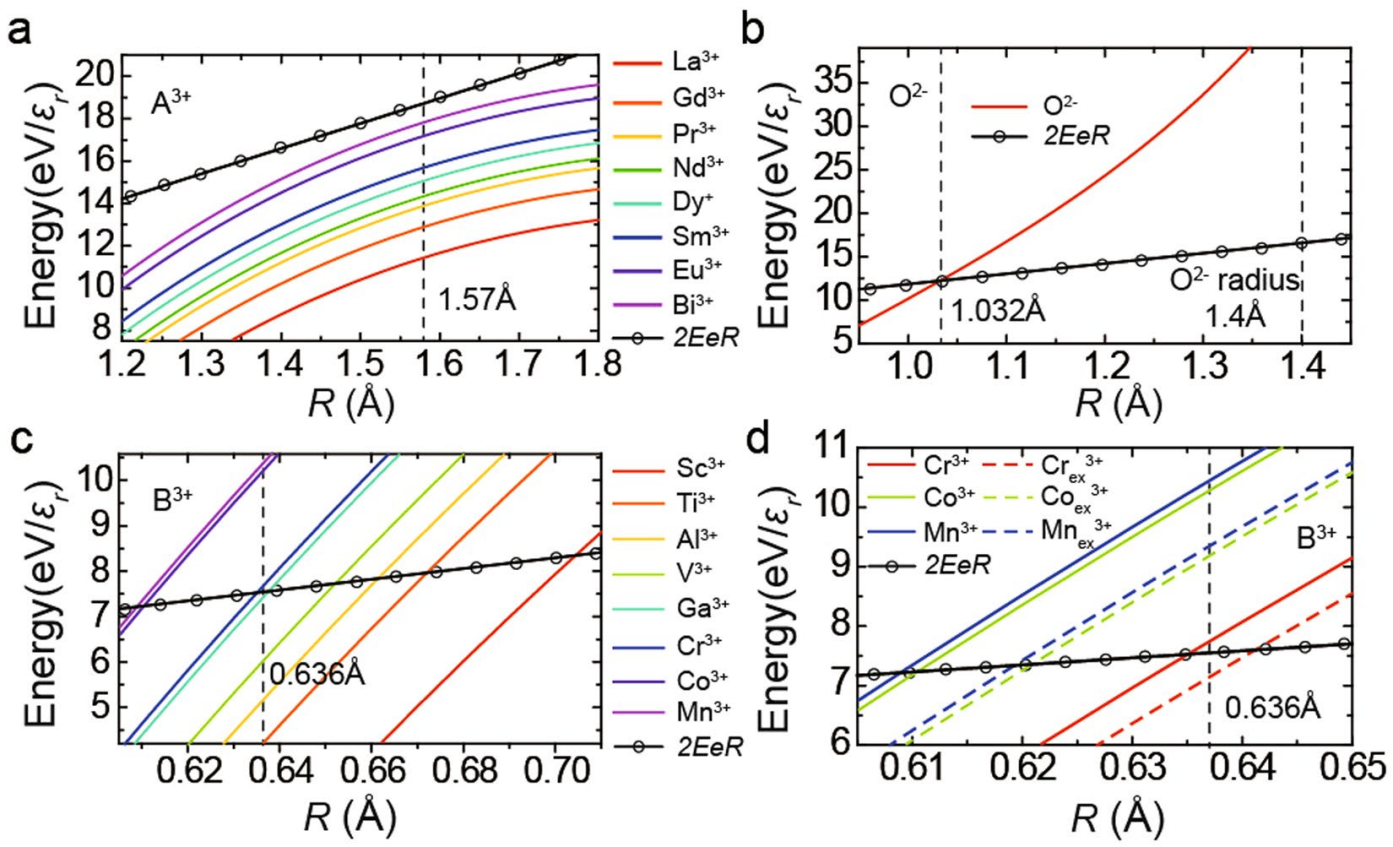
Simulation results for the two sides of Eq. (5) ([001] direction) as a function of *R*. (**a–c**) The situation for A^3+^, O^2−^, and B^3+^ IPWs. (**d**) The two sides of Eq. (5) for Cr^3+^, Co^3+^, Mn^3+^ (solid lines) and Cr^3+^_ex_, Co^3+^_ex_, Mn^3+^_ex_ (dashed lines). Cr^3+^_ex_, Co^3+^_ex_ and Mn^3+^_ex_ are the IPW depth when considering the influence of lattice expansion. The IPW depths for different ions are color-coded curves, and the energy increase (*2EeR*) is the black curve with circles. The dashed curves in (**a**,**c**), and (**d**) are the value of *R*. In (**b**), the dashed curves indicate the ion radius of O^2−^ (1.4 Å) and *R* (1.032 Å) at the crossing point between the IPW depth and energy change curves.

Equation (5) is independent of *ε*_*r*_ and *T* is the only variable factor for different ions, so *T* of A or B site cations (*T* = *I*_3_ for A^3+^ or B^3+^ cations) is the most important factor that determines the formation of 2DEG as shown in Fig. 4(a). *R* is the only unknown factor, which can be indirectly determined experimentally. To simplify our model, we assume that ions at the same sites (A, B, or O^2−^) have the same *R*. For *ε*_*r*_ = 1, the left side of Eq. (5) is the energy increment (equivalent to *2EeR*), which is ion-independent for the same-site-ions, while the right side of Eq. (5) is the IPW depth of different ions. To obtain *R*, we plot the two sides of Eq. (5) as a function of *R*, for A site ions, B site ions, and O^2−^ ions in the [001] direction (Fig. 5). 2DEG was discovered in LaGaO_3_/STO^17,44^, NdGaO_3_/STO^44^, NdAlO_3_/STO^44^, PrAlO_3_/STO^44^, LaVO_3_/STO^45^, LaTiO_3_/STO^46,47^, DyScO_3_/STO^48^, SmTiO_3_/STO^52^ and GdTiO_3_/STO^49^, but it was not observed in LaCrO_3_/STO^16,50^, LaMnO_3_/STO^17^, BiMnO_3_/STO^51^, and LaCoO_3_/STO. For all types of ions in these films, if the IPW depth (the colored curves in Fig. 5) is larger than the energy increment (the black curve in Fig. 5) for a certain value of *R*, no charge can escape from the IPW, and 2DEG will not form at the interface. Otherwise, 2DEG will form. It should be noted that, because here our model only focuses on the perovskite A^3+^B^3+^O_3_/STO interfaces, the other kinds of interfaces cannot be included, such as gamma-Al_2_O_3_/STO^53^, CaZrO_3_/SrTiO_3_^54^ and LSAT/STO^55^.

For B site cation IPWs, we observed that Mn^3+^, Co^3+^, Cr^3+^ have deeper IPWs than others, while Ga^3+^ and Cr^3+^ have very similar IPW depths (Fig. 5(c)). The trapping radius *R* should be located between the intersections of the IPW depth and energy increment, for Ga^3+^ and Cr^3+^ (Fig. 5(c)). In this article, we set *R* = 0.636 Å for B cite IPWs. For the A site cation IPWs, we found that the IPW depth are always less than the energy increment (Fig. 5(a)). This indicates that B site cation IPWs are stronger and play a more important role than A site cations in 2DEG formation. This is consistent with our findings (Fig. 4(a)). Here, we temporarily set *R* = 1.58 Å; at this value of *R*, the IPW depth and the energy increment are the most similar for all of the listed cations. The curves of O^2−^ IPWs considering Eq. (5) in the p-type heterojunction are plotted in Fig. 5(b). Note that holes in the p-type heterojunctions can reach the interface only if *R* is below 1.032 Å. But, the *R*-value of the O^2−^ IPW should be larger because the radius of O^2−^ (1.4 Å^29^) is significantly larger. This result suggests that the O^2−^ IPW is too deep and p-type 2DEG cannot form as have been found^1^. We also calculated the O^2−^ IPW in the [110] and [111] directions, and found that, theoretically, p-type 2DEG may form at the (111) interface (Fig. S5).

### The origin of 2DEG at LCO/STO interfaces

Inspired by the lattice enhancement of the LCO film (Fig. 1(d)), we studied the influence of the lattice constant on *eU*_0_(*R*)/*ε*_*r*_. We found that *eU*_0_(*R*)/*ε*_*r*_ increases with increasing the lattice constant (Fig. S7(c)), decreasing the IPW depth *eU*. Nevertheless, using our method to calculate *eU*_0_(*R*)/*ε*_*r*_, we found that oxygen vacancies cannot directly influence *eU*_0_(*R*)/*ε*_*r*_ because the electrons generated by oxygen vacancies have the same influence on *eU*_0_(*R*)/*ε*_*r*_ as O^2−^ ions. For example, the decrease in *eU* of LCO was 0.6 eV/*ε*_*r*_ when the lattice parameter was 3.957 Å (at 8 × 10^−7^ Torr) (Fig. 1(d)). This is sufficiently large for 2DEG formation at the LaCrO_3_/STO interface (Cr^3+^_ex_ in Fig. 5(d)), as the IPW depth of Cr^3+^ is near the critical value (~7.5/*ε*_*r*_ eV). Apparently, oxygen vacancies and the induced lattice expansion are the keys to the formation of 2DEG in LCO/STO. But, for the other initially insulating interfaces, such as LaMnO_3_/STO and LaCoO_3_/STO, the change in the IPW depth caused by the lattice expansionwas relatively small compared with the required change of ~2.7 eV/*ε*_*r*_ when considering a lattice parameter of 4 Å (Co^3+^_ex_ and Mn^3+^_ex_ in Fig. 5(d)). Thus, we determined that an insulator-metal transition caused by the introduction of oxygen vacancies cannot occur for these heterojunctions.

It is worthy to notice that *eU* is gradually varying with *P*_*ox*_ because of the gradual change of LCO lattice constant with *P*_*ox*_ (Fig. 1(d)). So that, there does not exist a critical *P*_*ox*_ that the charge escaping from IPWs and the insulator-metal transition can happen. The interfacial conductivity and the probability of charges escaping from IPWs are gradually varying with the *P*_*ox*_. That is the reason for the gradual change of interfacial resistance (Fig. 1(a)) and why a charge transfer can happen under a relatively high *P*_*ox*_ and result in a band bending on STO side (Fig. 3(a)).

### The trapping radios

Using Eq. (4) and the as-determined *R*, the trapping ratios *k* were calculated for different ions (Table 1). The value of *n*_*i*_ is determined by the cation’s IPW (A or B) with the strongest trapping ability (highest *k*) in the ABO_3_ film. It is worthy to notice that the calculated *n*_*i*_ somewhat deviates from experimental values, because our calculations use the same *R*. For LaCrO_3_/STO, LaMnO_3_/STO, and LaCoO_3_/STO, Cr^3+^ IPW, Mn^3+^ IPW, and Co^3+^ IPW exhibit 100% trapping efficiency (Table 1); thus, 2DEG does not form. For LaAlO_3_ films, Al^3+^ IPW (*k* = 67.9%) is more efficient than La^3+^ IPW (*k* = 61.1%). The theoretical value of the interface charge density *n*_*i*_ is 1.05 × 10^14^/cm^2^ and it corresponds to 0.16 Ti^3+^/u.c.^2^. This is in agreement with experimental findings^19,22^. Since many electrons on the STO side are localized^19,56^, *n*_*s*_ is much lower than *n*_*i*_.

**Table 1:** The trapping ratio (*k*), for different ions.

**A** ^**3+**^	**La** ^**3+**^	**Gd** ^**3+**^	**Pr** ^**3+**^	**Nd** ^**3+**^	**Dy** ^**3+**^	**Sm** ^**3+**^	**Eu** ^**3+**^	**Bi** ^**3+**^
*k*(%)	60.9	68.7	74.0	76.5	80.4	83.7	91.7	95.2
**B** ^**3+**^	**Sc** ^**3+**^	**Ti** ^**3+**^	**Al** ^**3+**^	**V** ^**3+**^	**Ga** ^**3+**^	**Cr** ^**3+**^	**Co** ^**3+**^	**Mn** ^**3+**^
*k*(%)	19.0	55.3	67.9	79.4	97.9	100	100	100

### The influence of substitution layers on LAO/STO interfaces

**Figure 6:**
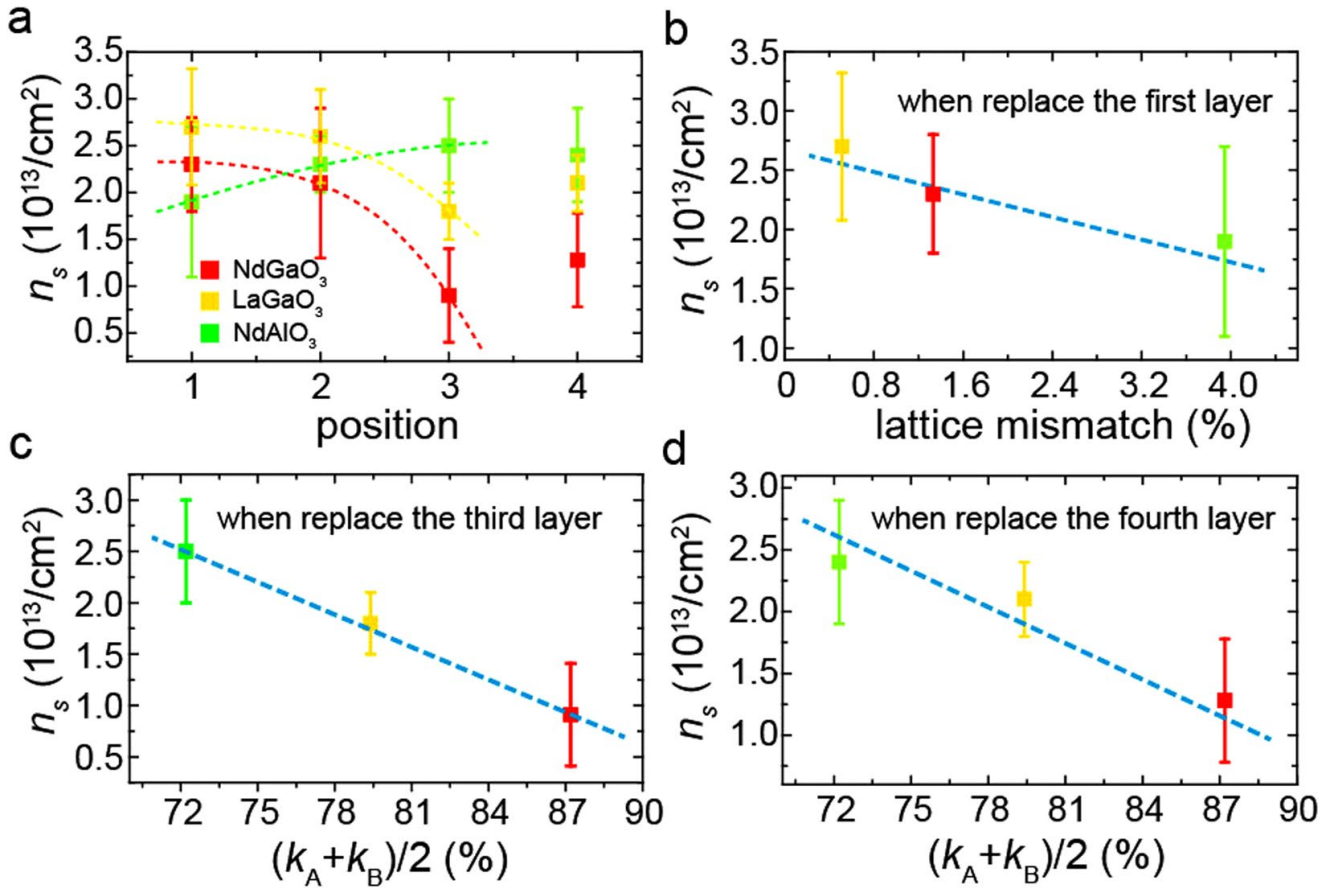
Influence of substitution layers on *n*_*s*_. (**a**) The influence of substitution oxide layers on *n*_*s*_. (**b–d**) The relationships between *n*_*s*_ and substitution oxides, for replacing the first (**b**), third (**c**), and fourth (**d**) unit layers. The parameters *k*_A_ and *k*_B_ are *k* (trapping percentage, Eq. (4)) of A site and B site cations of the substitution layers. (*k*_A_ + *k*_B_)/2 is the average *k* value.

The above discussion suggests that the IGSs in these films are a key factor deciding the formation and properties of 2DEG. Thus, by substituting some unit layers of a film with a heterojunction with 2DEG by other perovskite oxides, it could be possible to modulate the properties of 2DEG. Here, we replaced one unit layer of LAO(4 u.c.)/STO films with NdGaO_3_, LaGaO_3_, and NdAlO_3_. As expected, such substitutions affected *n*_*s*_. A strong relationship between *n*_*s*_ and substitution layers was found (Fig. 6).

When the first unit layer (closest to the interface) was replaced, *n*_*s*_ depended on the lattice mismatch between the substitution oxide and the substrate (Fig. 6(b)), in agreement with previous results^57,58^. The influence of the substitution layer on *n*_*s*_ when the third and fourth layers were replaced can be explained in terms of the average *k* values of A site and B site cation IPWs (Fig. 6(c,d)). We found that *n*_*s*_ is inversely proportional to the average *k*, as our model predicted (Eq. (3)). The effects of IPWs on the fourth and third layers were more pronounced than for other layers, because the former are the first and second layers, correspondingly, which the transferred electrons pass through owing to the 4 u.c. critical thickness of charge transfer. Interestingly, this result shows that A site and B site cation IPWs play the same role when only one unit layer is considered; yet, this is different from thick films. The influence of the substitution layer on *n*_*s*_ when the second layer is replaced is the mixture of the effects of lattice mismatch and IPWs (Fig. 6(a)). Generally, some oxygen vacancies will form in films during film growth due to the low *P*_*ox*_ and the high temperature. They can induce IPWs in films and influence the formation of 2DEG. Also, oxygen vacancies can form in STO substrates during films growth due to the oxygen diffusion, the bombardment by the plasma plume and so on^30^. By applying the ion trapping model to substrates (Fig. S6), we suggest that the IPWs induced by oxygen vacancies can give rise to IGSs and electron localization in substrates as have been found^34–37^. These results suggest that the IGS is a widespread and important factor in the formation of 2DEG, and we suggest that if the film has a perfect crystalline quality, a theoretical *n*_*s*_ may be achieved.

## Conclusions

We studied IGSs in films and found that these states are necessary for obtaining insulating interfaces with polar discontinuity. Oxygen vacancies should be necessary for the formation of IGSs in films. We propose the ion trapping model to explain how the IGS works. Our model is simple and phenomenological, defects, lattice distortion and some other factors are neglected. We think our research is a simple try, a deep theoretical research should be performed in future. Although our model is simple, it matched the experiments very well. Combining the polar catastrophe model and our model, the origin of interfacial 2DEG can be clearly understood. The 2DEG formed at the initially insulating LCO/STO interface and the influence of the substitution layer on *n*_*s*_ can also be explained.

## Methods

### Sample growth

**Figure 7:**
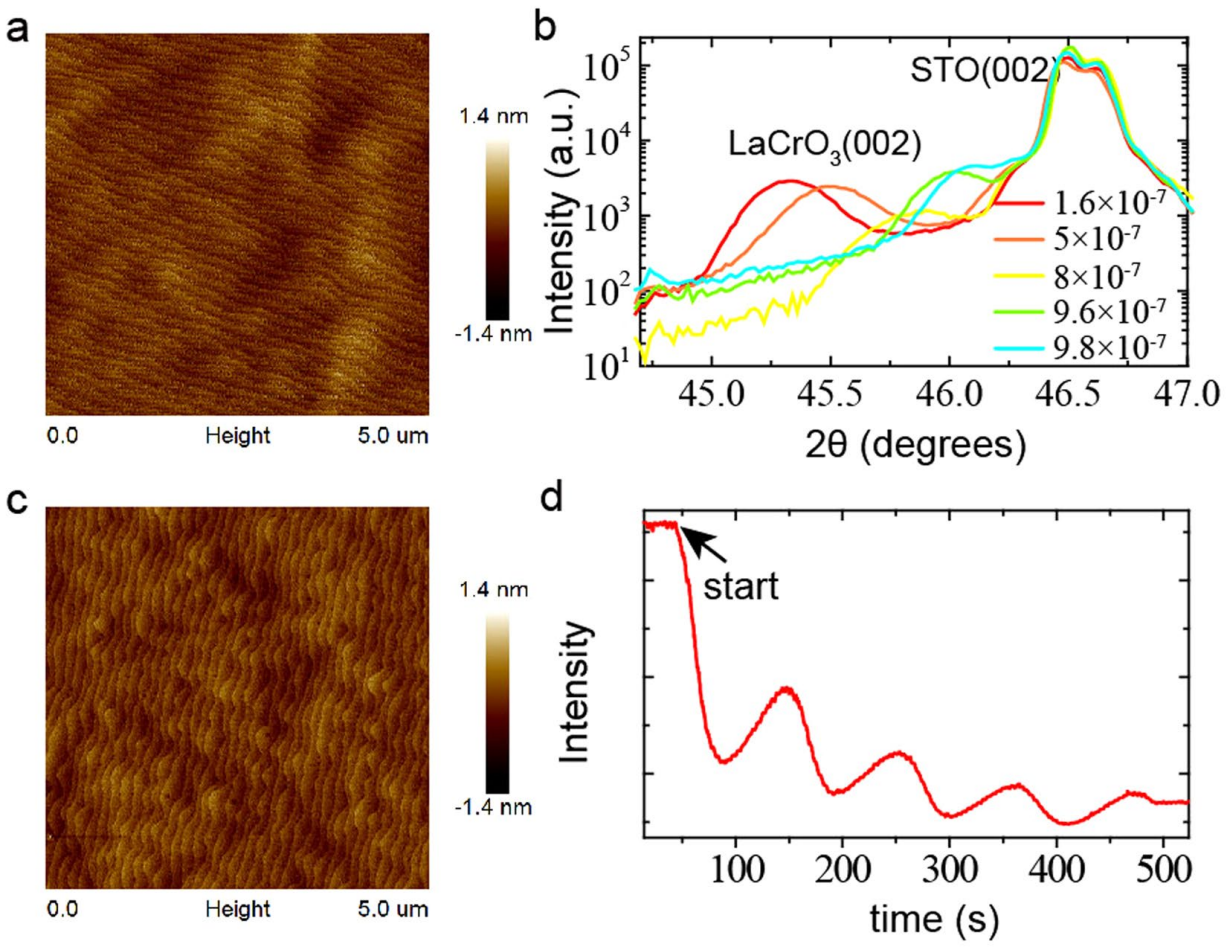
(**a**) AFM image of SrTiO_3_ substrates. (**b**) XRD patterns of LCO/STO (001) heterojunctions prepared in different growth oxygen pressure (Torr). (**c**) AFM image of the LaCrO_3_ film grown on (001) SrTiO_3_ in a partial *P*_*ox*_ of 6 × 10^−8^ Torr. (**d**) RHEED intensity oscillations of the specularly reflected beam for the growth of LaCrO_3_ (4 u.c.).

Films of LaCrO_3_, LaCoO_3_, LaMnO_3_, and SrTiO_3_ were grown at 830 °C, at different oxygen partial pressures, using pulsed laser deposition with laser energy of 0.9 J/cm^2^ and frequency of 1 Hz. The film growth was monitored using reflection high energy electron diffraction (RHEED). The substrate was TiO_2_-terminated (001) SrTiO_3_. AFM images of the substrate before deposition and of the LaCrO_3_ film are shown in Fig. 7(a,c). The XRD patterns of the films (Fig. 7(b)) indicating that the films were well epitaxial with (001) single phase character. We also found the lattice constant of the LaCrO_3_ film increases with decreasing oxygen partial pressure during growth (Fig. 7(b)).

### Program details

We coded a computer program to calculate the potential energy generated by different ions in a finite volume, given the distance (*r*) between the charge (one electron or hole) and IPW with *ε*_*r*_ = 1. The absolute value of the summation for all ions was *eU*_0_(*r*). In the calculation, we considered a volume of 1 × 10^6^ u.c.^3^ with the lattice constant of SrTiO_3_ (3.905 Å). Our calculations show a convergence trend with the increase in volume (Fig. S3), indicating that our simulation is coarse but functional. The arrangement of ions was different for different crystallographic directions; hence, *eU*_0_(*r*) was anisotropic (Fig. S4). The calculation method of our simulation was as follows:

$$\begin{array}{rcl}\frac{e{U}_{0}({r}_{[h,k,l]})}{{\varepsilon }_{r}} & = & {\sum }^{}\frac{3{e}^{2}}{4\pi {\varepsilon }_{r}{\varepsilon }_{0}|{\mathop{r}\limits^{\rightharpoonup }}_{{A}^{3+}}-{\mathop{r}\limits^{\rightharpoonup }}_{[h,k,l]}|}\\  &  & +{\sum }^{}\frac{3{e}^{2}}{4\pi {\varepsilon }_{r}{\varepsilon }_{0}|{\mathop{r}\limits^{\rightharpoonup }}_{{B}^{3+}}-{\mathop{r}\limits^{\rightharpoonup }}_{[h,k,l]}|}-{\sum }^{}\frac{2{e}^{2}}{4\pi {\varepsilon }_{r}{\varepsilon }_{0}|{\mathop{r}\limits^{\rightharpoonup }}_{{O}^{2-}}-{\mathop{r}\limits^{\rightharpoonup }}_{[h,k,l]}|}\end{array}$$

Here, ${\mathop{r}\limits^{\rightharpoonup }}_{[h,k,l]}$ $(|{\mathop{r}\limits^{\rightharpoonup }}_{[h,k,l]}|=r)$ is the position of the charge to the IPW, in the [h, k, l] direction. The distances $|{\mathop{r}\limits^{\rightharpoonup }}_{{A}^{3+}}-{\mathop{r}\limits^{\rightharpoonup }}_{[h,k,l]}|$, $|{\mathop{r}\limits^{\rightharpoonup }}_{{B}^{3+}}-{\mathop{r}\limits^{\rightharpoonup }}_{[h,k,l]}|$ and $|{\mathop{r}\limits^{\rightharpoonup }}_{{O}^{2-}}-{\mathop{r}\limits^{\rightharpoonup }}_{[h,k,l]}|$ are the distances between the positions of A^3+^, B^3+^, and O^2−^ ions (${\mathop{r}\limits^{\rightharpoonup }}_{{A}^{3+}}$, ${\mathop{r}\limits^{\rightharpoonup }}_{{B}^{3+}}$ or ${\mathop{r}\limits^{\rightharpoonup }}_{{O}^{2-}}$) and the position of charge to the IPW $({\mathop{r}\limits^{\rightharpoonup }}_{[h,k,l]})$. Our simulation considers ions from the film’s bulk. Surface states of the film and ions of the substrate (SrTiO_3_) are neglected because they are expected to have a weak and indirect influence on eU_0_ and IPWs. Film thickness was not taken into consideration.

## Electronic supplementary material

Supplementary Information: Influence of In-Gap States on the Formation of Two-Dimensional Election Gas at ABO3/SrTiO3 Interfaces
