# Supplementary material for: Influence of In-Gap States on the Formation of Two-Dimensional Election Gas at ABO3/SrTiO3 Interfaces
Source: Sci Rep. 2018 Jan 9;8:195. doi: 10.1038/s41598-017-18583-5 (PMC5760580; doi:10.1038/s41598-017-18583-5)
Supplement: Supplementary file 1 — Supplementary Information: Influence of In-Gap States on the Formation of Two-Dimensional Election Gas at ABO3/SrTiO3 Interfaces [file 41598_2017_18583_MOESM1_ESM.pdf]

Supplementary Information:
Influence of In-Gap States on the Formation of Two-Dimensional Election Gas at $ABO_3/SrTiO_3$ Interfaces

Cheng-Jian Li,[1] Hong-Xia Xue,[1] Guo-Liang Qu,[1] Sheng-Chun Shen,[1] Yan-Peng Hong,[1] Xin-Xin Wang,[1] Ming-rui Liu,[1] Wei-min Jiang,[1] Petre Badica,[2] Lin He,[1] Rui-Fen Dou,[1] Chang-Min Xiong,[1] Wei-ming Lü,[3] Jia-Cai Nie[1*]

[1] *Department of Physics, Beijing Normal University, Beijing, 100875, China*

[2] *National Institute of Materials Physics, Atomistilor 405A, Magurele, Ilfov, 077125, Romania*

[3] *Condensed Matter Science and Technology Institute, Harbin Institute of Technology, Harbin, 150001, China*

Derivation process:

As described in the manuscript, the electric field in polar-non-polar heterojunctions has two main components ($E = E_p + E_i$), one owing to polar discontinuity and the other owing to the charges at the interface (eq.(1)). The IPW depth eU can be described as eq.(2)

$$E = E_p + E_i = \frac{n_p e}{\varepsilon_r \varepsilon_0} - \frac{n_i e}{\varepsilon_r \varepsilon_0} = \frac{(n_p - n_i)e}{\varepsilon_r \varepsilon_0} \qquad \text{eq.(1)}$$

$$eU = \frac{T}{\varepsilon_r} - \frac{eU_0(R)}{\varepsilon_r} \qquad \text{eq.(2)}$$

n_p is the theoretical interfacial charge density in the polar catastrophe model, which is ~$3.28\times10^{14}/cm^2$ for $A^{3+}B^{3+}O_3$/STO(001) heterojunctions. n_i is the real interface charge density.

As described in the manuscript, $2EeR = eU$ is the conduction that the charge escaping from IPW to interface stops. The E is described as eq.(1) and eU is described as eq.(2). Combine eq.(1), eq.(2) and $2EeR = eU$, we have:

$$\frac{2(n_p - n_i)e^2 R}{\varepsilon_r \varepsilon_0} = \frac{T}{\varepsilon_r} - \frac{eU_0(R)}{\varepsilon_r}$$

Then

$$n_i = n_p - \frac{T - eU_0(R)}{2e^2 R}\varepsilon_0$$

It can be written as

$$n_i = n_p\left(1 - \frac{T - eU_0(R)}{2e^2 R n_p}\varepsilon_0\right)$$

Here we set $\boldsymbol{k = \frac{T - eU_0(R)}{2e^2 R n_p}\varepsilon_0}$ (eq.(4)) we have:

$$n_i = n_p(1 - k) \qquad \text{eq.(3)}$$

k is the percentage trapping of electrons.

TABLE S1: The ionization energy (I) or the electronic affinity (A) (KJ/mol) of different ions[1].

Cations	I_3	Cations	I_3	Cations	I	Anions	A_2
Sc^{3+}	2389	La^{3+}	1850	Ti^{4+}	4175 (I_4)	O^{2-}	798
Ti^{3+}	2653	Gd^{3+}	1990	Sr^{2+}	1064 (I_2)		
Al^{3+}	2745	Pr^{3+}	2086				
V^{3+}	2828	Nd^{3+}	2130				
Ga^{3+}	2963	Dy^{3+}	2200				
Cr^{3+}	2987	Sm^{3+}	2260				
Mn^{3+}	3248	Eu^{3+}	2404				
Co^{3+}	3232	Bi^{3+}	2466				

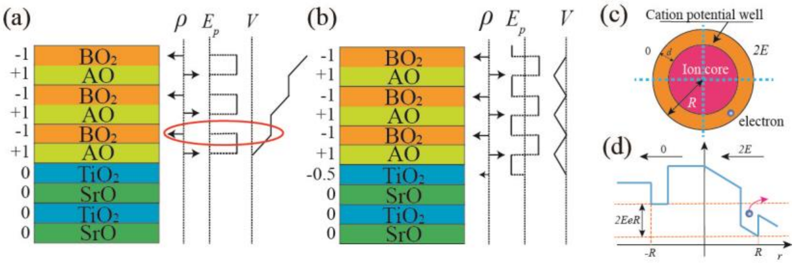

FIG. S1. (Color online) (a,b) The schematic diagram of the polar catastrophe model for n-type interface (the STO substrate is TiO_2-terminated). This model postulates that $A^{3+}B^{3+}O_3$ is polar with alternating sheets of positive and negative charge in the (001) direction and STO is not polar. To accommodate the diverging potential caused by the polar discontinuity at the $A^{3+}B^{3+}O_3$/STO interface (a), about half an electrons transfer to the interface and form 2DEG (b). (c) Schematic diagram of IPW and the electrical field in the region marked by the red ellipse in (a). The blue vertical dashed line is the electrical field boundary between 0 and *2E* states. (d) The *r*-dependence of the potential energy for one electron along the horizontal dashed line from (c), when *2EeR*>*eU*.

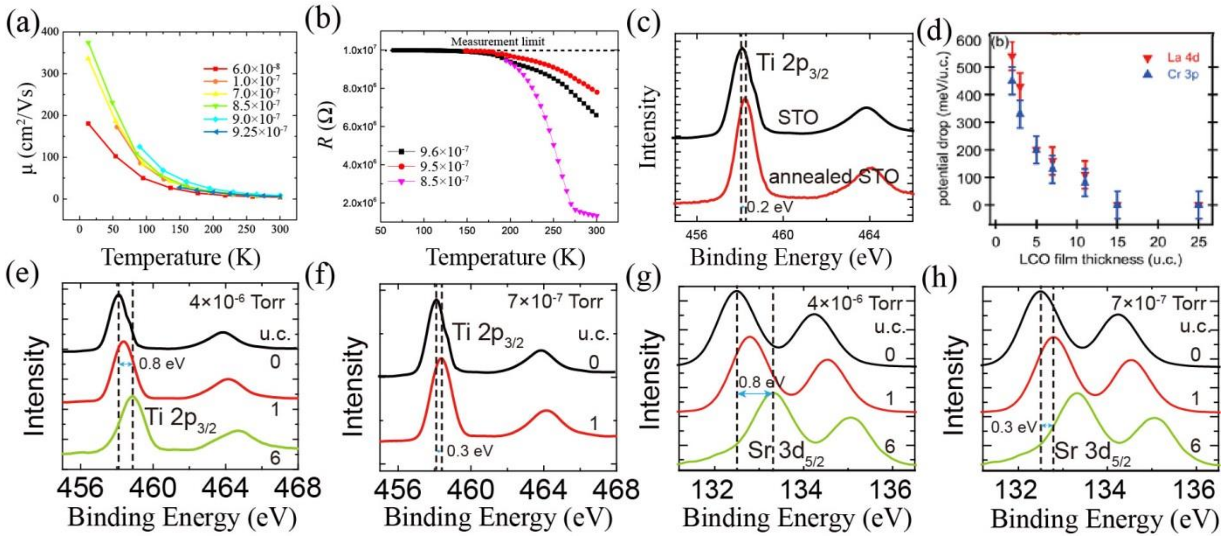

FIG. S2. (Color online) Temperature dependence of: (a) the charge mobility of the $LaCrO_3/SrTiO_3$ interface and (b) the resistance of the $LaCrO_3$ film grown for different oxygen pressure (Torr). (c) The core level spectra of Ti 2p of bulk STO and annealed STO (annealed in 2×10^{-7} Torr for 2 hours, it is not conductive), an core level shift was found. (d) The binding energy determined by the core levels of La and Cr for different LCO thickness[2]. (e-h) The core level spectra of Ti $2p_{3/2}$ (e,f) and Sr $3d_{5/2}$ (g,h) for LCO/STO with different thicknesses of LCO when the film grown at a low oxygen pressure (f,h) and high oxygen pressure (e,g). As showed in (d-h), the core level shifts of A-site and B-site cations are similar.

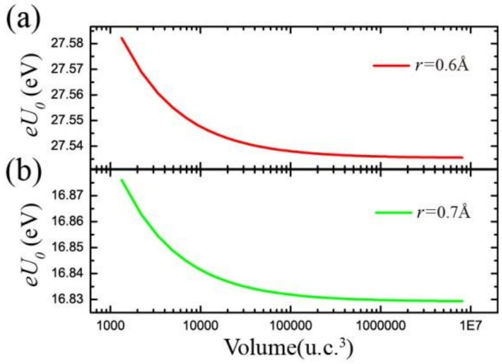

FIG. S3. (Color online) Volume dependence of the (charge normative) background potential energy eU_0 calculated in the [001] direction for the B^{3+} cation when r=0.6 Å and r=0.7 Å. Results show a convergence trend with the film's volume. This indicates that the eU_0-value for a volume of 1×10^6 u.c.[3] can be taken as the actual value.

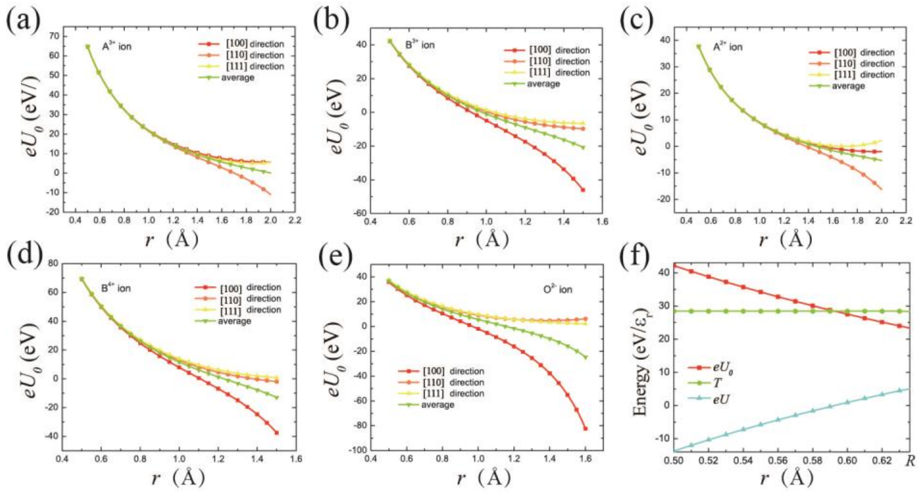

FIG. S4. (Color online) (a-e) Background potential eU_0 of different ions in the ABO_3 film on different directions and the average value mediated over all investigated directions. (f) The r dependence (for $r < R$, i.e. where IPW is meaningful) of eU_0 (red), T (green) and $eU = T - eU_0$ (blue) of Al^{3+} IPW. R is the trapping radius of IPW. Noteworthy, eU is maximum when $r = R$. The Al^{3+} IPW is presented as an example since other ions show a same behavior.

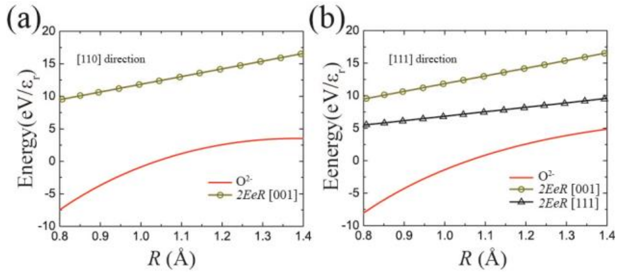

FIG. S5. (Color online) Simulated curves of the two sides of Eq. (5) for O^{2-} IPW in the ABO_3 film along (a) [110] and (b) [111] directions. $2EeR$ [001] and $2EeR$ [111] represent the energy increase on the indicated directions. The IPW depth is smaller than the energy increase in [111] direction: the p-type 2DEG may occur theoretically. However, it is very hard to form p-type 2DEG, because oxygen vacancies in film will generate a large number of electrons, the recombination between holes and these electrons will prevent the formation of p-type 2DEG.

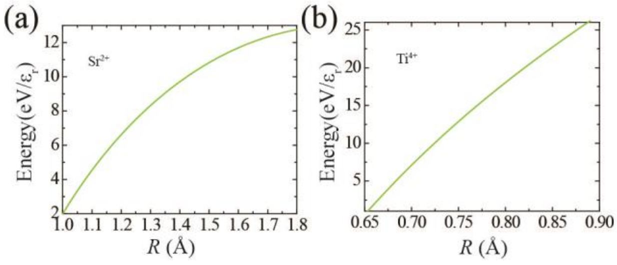

FIG. S6. (Color online) The IPW depth of Sr^{2+} (a) and Ti^{4+} (b) in substrate. The IPWs in substrate should concentrate near the interface because here have a larger lattice distortion and high defects density which are necessary for IPWs formation. IPW states are localized states and resulting in a lower n_s comparing with n_i. Because substrate has much fewer defects than film, the density of IPWs in substrate should be very low, so that the IGS in substrate is hardly detected. The low IPWs density is also essential for 2DEG formation. If IPWs density at STO side is too high, all transferred electrons will be localized and n_s will be 0.

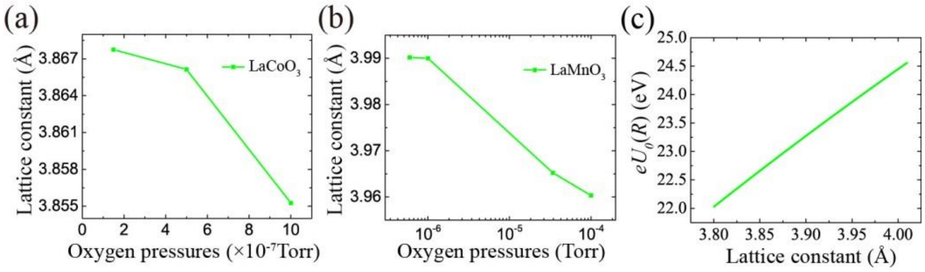

FIG. S7. (Color online) The lattice constant estimated from XRD for (a) (001)$LaCoO_3$ and (b) (001)$LaMnO_3$ films vs. the growth oxygen partial pressure. (c) The lattice constant dependence of $eU_0(R)$.

1. Catherine E. Housecroft, Alan G. Sharpe. *Inorganic Chemistry*. (2012).

2. Chambers, S. A. *et al.* Band Alignment, Built-In Potential, and the Absence of Conductivity at the LaCrO3/SrTiO3(001) Heterojunction. *Phys. Rev. Lett.* **107,** 206802 (2011).
